# 

**Published:** 1902-01

**Authors:** 


### CONTENTS.

ORIGINAL COMMUNICATIONS. PAGE

Faulty Environment. By E. K. Wedelstaedt, St. Paul, Minn. . . . . . . 1
An Unusual Operation. By W. R. Howard, D.D.S., Newport, R. I. . . . . . 9
Helps to Success in Practice. By. George L. Parmele, M.D., D.M.D., '70, Hartford, Conn. . . . . . . 11
"Foreign Observations." By Frederick Bradley, D.M.D., Newport R. I. . 15
A Few Extracts from "Fox and Harris" in Contrast. By Henry C. Spencer, D.M.D., '97, Newton, Mass. . . . . . . 21
The Control of our Patients. By Henry A. Kelley, D.M.D., '88, Portland, Me. 26

REVIEWS OF DENTAL LITERATURE . . . . . . . 31

REPORTS OF SOCIETY MEETINGS.

American Academy of Dental Science . . . . . . . 33
Harvard Dental Alumni Association . . . . . . . 40
International Dental Federation: Meetings of Executive Council, Subcommittee, and International Commission of Education . . . . . . . 43

EDITORIAL.

What is the True Standard of Mental Training? . . . . . . . 54
Is Spelling an Important Factor in Examination? . . . . . . . 56
Dr. R. W. Morgan retired . . . . . . . 59

BIBLIOGRAPHY . . . . . . . 59

OBITUARY.

Charles J. Essig, M.D., D.D.S., . . . . . . . 61
Tennessee Dental Association—Resolutions of Respect to Dr. W. H. Morgan . 65
In Memoriam . . . . . . . 66

CURRENT NEWS.

Northern Illinois Dental Society . . . . . . . 66
Southern Branch National Dental Association . . . . . . . 67
New York Odontological Society . . . . . . . 67
Pennsylvania Association of Dental Surgeons . . . . . . . 68

